# Supplementary material for: Cholesterol Attenuates the Pore-Forming Capacity of CARC-Containing Amphipathic Peptides
Source: Int J Mol Sci. 2025 Jan 10;26(2):533. doi: 10.3390/ijms26020533 (PMC11765261; doi:10.3390/ijms26020533)
Supplement: Supplementary file 1 [file ijms-26-00533-s001.zip › ijms-3375515-supplementary.pdf]

Supplementary material for

Cholesterol Attenuates the Pore-Forming Capacity of CARC-Containing Amphipathic Peptides

Ilya P. Oleynikov, Alexander M. Firsov, Natalia V. Azarkina* and Tatiana V. Vygodina

* Correspondence author: azarkina@yahoo.com

Table S1. Kinetic parameters of calcein release induced by peptides A4, P4 and A1. Liposomes did not contain (control) or contained membrane cholesterol. See Figure 3 in the Main text. Experimental curves were approximated by the function $y= k(1-\exp(-x/\tau))+ const$, where τ is the characteristic time, k is the amplitude. In cases where the process was not described by a single component, the presence of additional phases is noted. Presumably present phases are indicated in brackets.

Peptide	Liposomes	Concentration of the peptide, μM	τ, s	Amplitude, %	Other phases
A4	control	0.2	163.6 ± 6.0	100	
		+0.3	21.2 ± 0.02	27	fast
	cholesterol	0.2	205.4 ± 126	100	
		+0.3	147.4 ± 5.1	7.2	fast and slow
P4	control	1.0	3.4 ± 0.005	100	
	control	0.5	233.4 ± 2.2	100	
	control	0.2	395.6 ± 28.7	43	fast
		+0.3	45 ± 0.03	41	fast
	cholesterol	0.2	40 ± 0.18	100	
		+0.3	190.7 ± 4.6	63	fast
A1	control	5	2.0 ± 0.005	100	
	control	1	14.4 ± 0.6<br>230.7 ± 5.8	ca. 30<br>ca. 70	
	control	0.2	20.8 ± 4.3	100	
		+0.3	94.6 ± 5.2	≤ 100	(fast)
		+0.5	84.9 ± 2.5	≤ 100	(fast and slow)
	cholesterol	0.2	230 ± 21	≤ 100	(fast)
		+0.3	201.4 ± 8.9	≤ 100	(slow)
		+0.5	170.6 ± 6.7	≤ 100	(fast and slow)

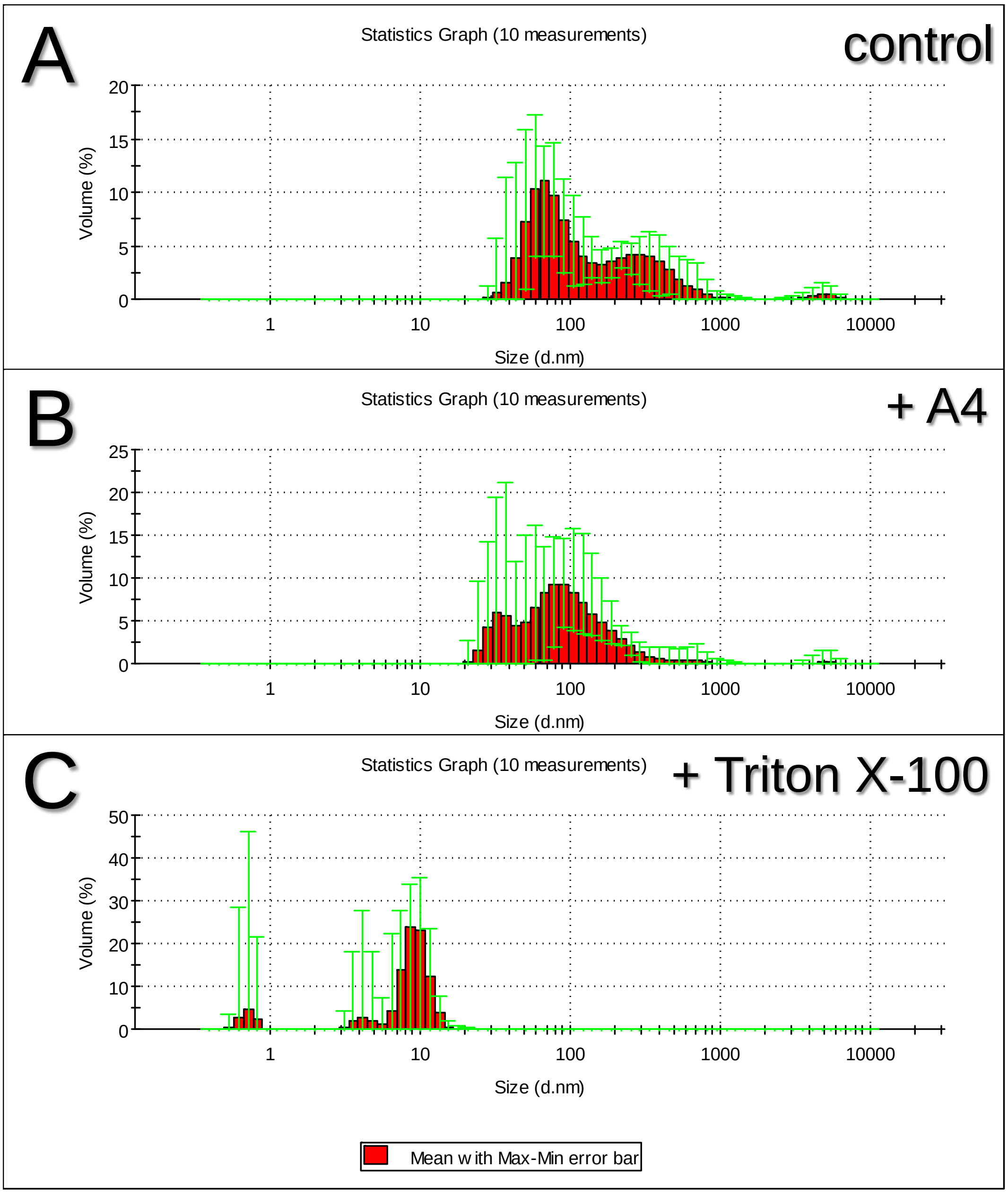

Figure S1. Peptide A4 does not disrupt the membrane of asolectin liposomes.

Size distribution of asolectin liposomes. Particle size in suspension was estimated by dynamic light scattering. Liposomes were diluted 1:100 in 100 mM KCl, 25 mM HEPES/Tris, pH 8.0. Measurements were performed on a Zetasizer Nano ZS device (Malvern Instruments, Malvern, Worcestershire, UK) at 25^0. Data are presented using Dispersion Technology Software version 5.0.

A – liposome suspension without additives;

B – 10 µM peptide A4 was added to the liposome suspension 3 minutes before the start of measurements;

C – 0.5% Triton X-100 was added to the liposome suspension 3 minutes before the start of measurements.

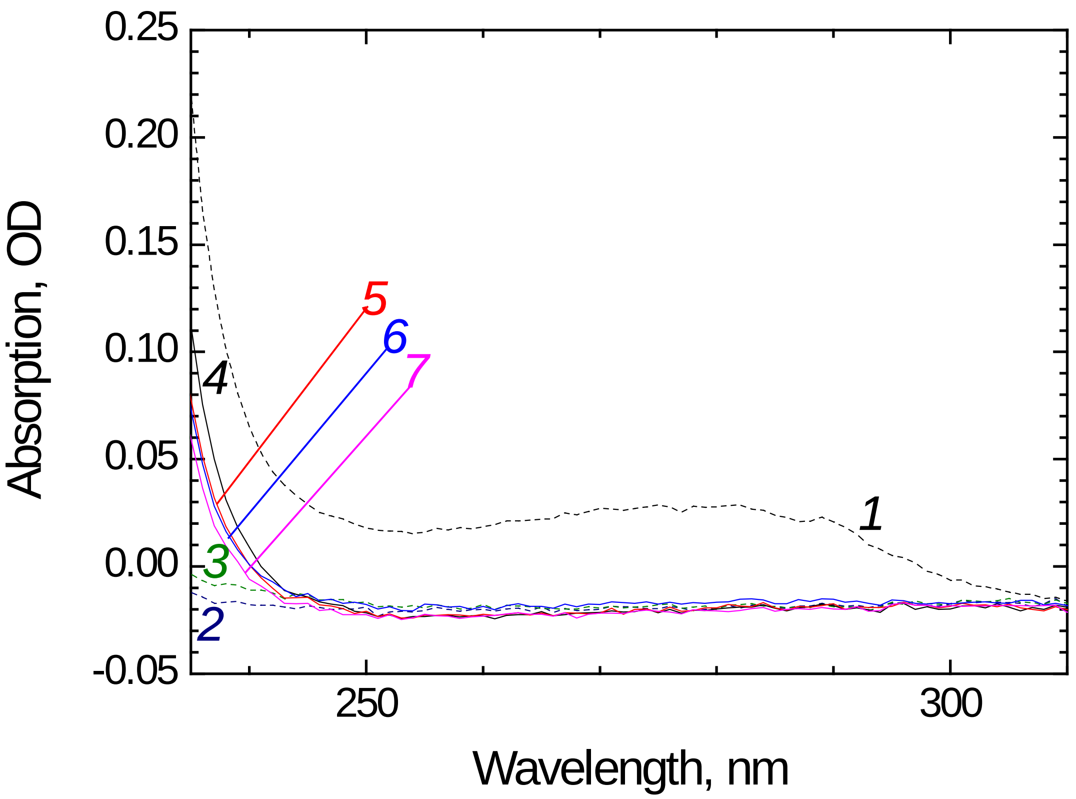

Figure S2. Membrane cholesterol does not prevent the sorption of peptide A4 on asolectin liposomes. Asolectin liposomes, either cholesterol-containing or cholesterol-free, were prepared in medium 1 (200 mM sucrose, 25 mM HEPES/Tris, pH 8.0) and transferred to medium 2 (100 mM KCl, 25 mM HEPES/Tris, pH 8.0) at a dilution of 1:100 or 1:25. Then, they were span down (50,000 g, 60 min) either in the presence of 10 μM peptide A4 or without additions. After centrifugation, optical absorption spectrum of supernatant was recorded. The spectra of the following samples are shown:

1 (black dash) – 10 μM peptide A4 in medium 2;

2 (navy dash) – control sample supernatant without peptide, liposomes without cholesterol (1:25);

3 (olive dash) – control sample supernatant without peptide, liposomes with cholesterol (1:25);

4 (black) – A4 sample supernatant, liposomes without cholesterol (1:100);

5 (red) – A4 sample supernatant, liposomes with cholesterol (1:100);

6 (blue) – A4 sample supernatant, liposomes without cholesterol (1:25);

7 (magenta) – A4 sample supernatant, liposomes with cholesterol (1:25).
